# The what, why and how of aromatase inhibitors: hormonal agents for treatment and prevention of breast cancer

**DOI:** 10.1111/j.1742-1241.2007.01587.x

**Published:** 2007-12

**Authors:** C J Fabian

**Affiliations:** Breast Cancer Prevention Center, Division of Clinical Oncology, Department of Internal Medicine, University of Kansas Medical Center Kansas City, KS, USA

## Abstract

The third-generation aromatase inhibitors (AIs) anastrozole, exemestane and letrozole have largely replaced tamoxifen as the preferred treatment for hormone receptor – positive breast cancer in postmenopausal women. Approximately 185,000 new cases of invasive breast cancer are diagnosed yearly, and at least half of these women are both postmenopausal and eligible for adjuvant therapy with AIs. In addition, AIs are currently being tested as primary prevention therapy in large randomised trials involving tens of thousands of women at increased risk for breast cancer. Given the volume of use, internists will increasingly see postmenopausal women who are taking or considering treatment with AIs. Physicians need to be able to: (i) briefly discuss the pros and cons of using a selective estrogen receptor modulator such as tamoxifen or raloxifene vs. an AI for risk reduction and (ii) recognise and manage AI-associated adverse events. The primary purpose of this review is to help internists with these two tasks.

**Review Criteria**

Expert opinion based on review of literature on relevant clinical trials.

**Message for the Clinic**

Both tamoxifen and AIs are effective for the adjuvant and neoadjuvant treatment of postmenopausal breast cancer; the optimal choice of drug is dependent on the characteristics of the patient and tumour. Adverse events with both drug classes are manageable. Adverse events associated with tamoxifen include increased risk of uterine cancers and thromboembolic events vs. an increased incidence of vaginal dryness, loss of libido, musculoskeletal pain and bone mineral density loss with AIs. Promising studies of AIs in the breast cancer prevention setting are ongoing.

## Introduction

**Figure 1:**
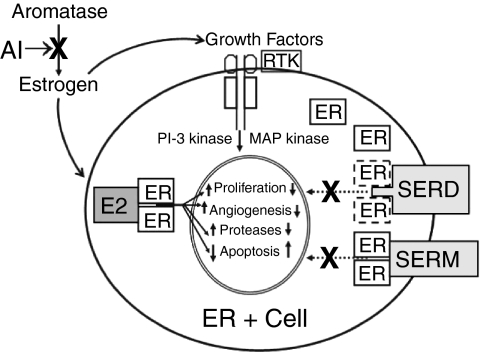
Schematic of metabolic pathways in an ER-positive cell that can be affected by AIs. The left side represents the active pathways and cellular responses under normal estrogen control. The right side depicts the blockade of pathways involving ERs and the resultant cellular responses. AI, aromatase inhibitor; E_2_, estradiol; ER, estrogen receptor; MAP, mitogen-activated protein; PI-3, phosphoinositide-3; RTK, receptor tyrosine kinase; SERD, selective estrogen receptor down-regulator; SERM, selective estrogen receptor modulator

Estrogen promotes the growth and survival of normal and cancerous breast epithelial cells by binding and activating the estrogen receptor (ER). The activated receptor in turn binds to gene promoters in the nucleus and activates many other genes responsible for cell division, inhibition of cell death, new blood vessel formation and protease activity. An increase in the proportion of cells that express ER is found at both the earliest stages of breast precancer and in approximately 70% of breast cancers (1). There are three ways in which estrogen-dependent processes important in the development and progression of the majority of breast cancers may be interrupted (Figure 1). The first is to interfere with the binding of estrogen to the ER and/or to the promoter elements of the genes it regulates. Selective ER modulators such as tamoxifen and raloxifene act in this manner. A second method is to reduce or eliminate ER expression. This is exemplified by fulvestrant, a selective ER down-regulator, which works by making less receptor available for binding to estrogen. The most direct means is to simply reduce the amount of estrogen by interfering with its production, via ovarian ablation in premenopausal women and use of aromatase inhibitors or inactivators (AIs) in postmenopausal women. Because of their effectiveness, AIs are quickly becoming the most frequently used antihormonal treatment for breast cancer in postmenopausal women. Further, AIs are now being tested in breast cancer prevention trials.

Aromatase inhibitors are not without adverse effects, which primarily stem from profound estrogen depletion. Many women will turn to their internists for advice about whether to take these drugs, as well as help in preventing and managing adverse events. The purpose of this article is to provide primary care physicians with a basic understanding of AIs to help facilitate these interactions.

## What is an aromatase inhibitor and how does it work?

**Figure 2:**
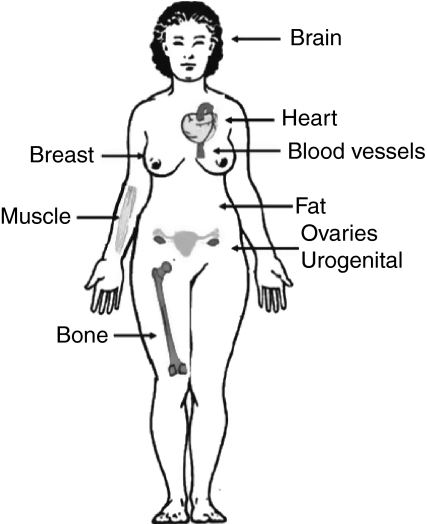
Schematic of organs with substantial aromatase activity

Aromatase inhibitors and inactivators interfere with the body's ability to produce estrogen from androgens by suppressing aromatase enzyme activity. Before menopause, ovarian aromatase is responsible for the majority of circulating estrogen and is exquisitely sensitive to changes in luteinising hormone (LH). Following menopause, aromatase in fat and muscle may be responsible for much of the circulating estrogen. Aromatase in highly estrogen-sensitive tissues, such as the breast, uterus, vagina, bone, brain, heart and blood vessels, provides local estrogen in an autocrine fashion (Figure 2). The aromatase gene promoter in breast tissue is less sensitive than the gene promoter in the ovary to fluctuations in LH but much more sensitive to increases in inflammatory cytokines. Circulating inflammatory cytokines increase with age, and breast tissue inflammatory cytokines increase with proliferative breast disease and breast cancer. Thus, it comes as little surprise that breast aromatase activity is increased in proliferative breast disease and many cases of breast cancer (2).

**Figure 3:**
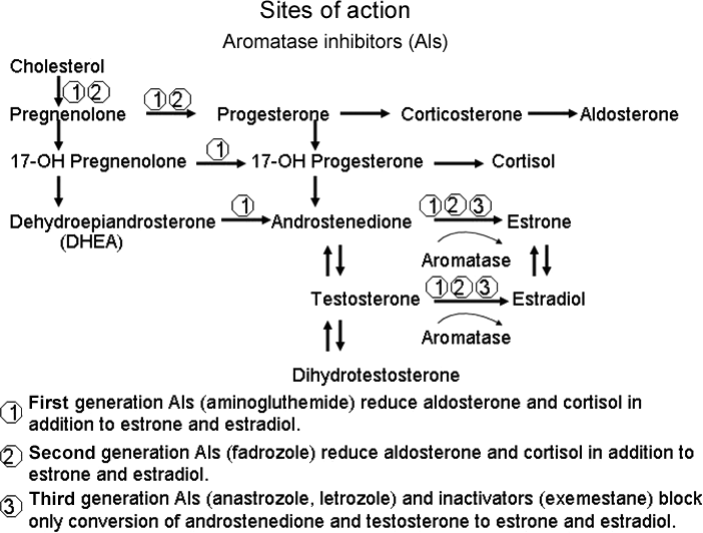
Metabolic pathways differentially targeted by aromatase inhibitors (AIs)

Three generations of AIs have been developed (Table 1) (3–8). Each successive generation has been associated with higher specificity for the aromatase enzyme (Figure 3), fewer adverse events, and greater suppression of aromatase activity. The utility of first- and second-generation AIs was limited by adverse events, such as rash, fatigue, dizziness, ataxia, nausea and vomiting, as well as by a lack of enzyme selectivity. Third-generation AIs are superior to earlier versions because they are associated with fewer adverse events and greater suppression of aromatase activity. There are two classes of third-generation AIs. Non-steroidal AIs reversibly bind to the aromatase enzyme and include anastrozole and letrozole. The steroidal AI exemestane binds to aromatase irreversibly. All third-generation AIs are administered orally on a daily basis. Adverse events include hot flushes, vaginal dryness, loss of libido, fatigue, arthralgias, joint stiffness and loss of bone mineral density with subsequent increased risk of fracture (9). In premenopausal women, AIs have a limited ability to reduce circulating estrogen. Unlike postmenopausal women, premenopausal women have a large amount of aromatase substrate present in the ovary. The exquisite sensitivity of the ovarian aromatase promoter to gonadotrophins, which increase dramatically after AI administration, makes AIs less effective in inhibiting ovarian estrogen production. Thus, AIs are generally not given to premenopausal women for breast cancer treatment without the addition of a medication to suppress the rise in gonadotrophins and subsequent increase in hormone levels (9).

**Table 1:** Efficacy of aromatase suppression by three generations of AIs

Drug	Dose	% Inhibition
**First generation**		
Aminoglutethimide (1,3)	1 g	91
**Second generation**		
Fadrozole (100)	2 mg	82
Vorozole (5)	1 mg	93
**Third generation**		
Letrozole (100,101)	2.5 mg	99
Anastrozole (100,102)	1 mg	97
Exemestane (100,103,104)	25 mg	98

AIs, aromatase inhibitors.

## Why do we need aromatase inhibitors?

For women with newly diagnosed hormone receptor positive ER+ cancers requiring systemic adjuvant therapy, 5 years of tamoxifen reduces the relative odds of recurrence by 40% and relative risk of death from breast cancer by 34% (10). At 15 years this equates to about a 12% absolute reduction in recurrence and a 9% absolute reduction in mortality, irrespective of nodal status. However, about a third of women diagnosed with ER-positive breast cancer will ultimately relapse despite adjuvant tamoxifen with or without chemotherapy (10). Women with hormone receptor-positive disease that has metastasised to organ sites distant from the breast almost always relapse following first-line antihormonal therapy with tamoxifen. More effective antihormonal treatment for tamoxifen-resistant tumours are needed.

There is some evidence suggesting a worse outcome with tamoxifen for women with ER-positive tumours that lack progesterone receptor (PgR), and/or exhibit overexpression of growth factor receptors such as human epidermal growth factor receptors 1and 2 (EGFR and HER-2/neu) (11,12). The observation that prolonged administration of tamoxifen may increase rather than decrease late recurrence rates (13) may be due to tamoxifen's ability to act as a partial estrogen agonist in breast tissue under conditions of growth factor receptor up-regulation, which commonly occurs after prolonged tamoxifen use (11,12). AIs appear to be more effective than tamoxifen in ER-positive tumours regardless of PgR or growth factor receptor status (14,15).

Treatment with AIs produce frequent and durable responses in postmenopausal women previously treated with tamoxifen or endocrine ablative surgery, and AIs are more effective than tamoxifen in producing responses and delaying progression in first-line treatment of metastatic disease (16). A recent meta-analysis concluded that in women with metastatic breast cancer, AIs show a survival benefit when compared with other endocrine therapy (17).

## How are aromatase inhibitors currently used?

The third-generation AIs are currently the preferred first-line treatment for metastatic hormone receptor-positive tumours and have all been approved by the US Food and Drug Administration for adjuvant use in postmenopausal women before or after surgery for ER-positive and/or PgR-positive breast cancer (18). Although anecdotal responses have been observed in women with ER- and PgR-negative tumours, in current clinical practice, only postmenopausal women with ER-positive and/or PgR-positive tumours are selected for treatment with AIs (9,16). There are several clinical studies evaluating the use of AIs in premenopausal women combined with ovarian suppression with a LH-releasing hormone (LHRH) analogue. AIs are generally not used off-label for premenopausal women except in special circumstances, such as prior tamoxifen failure or medical contraindications to tamoxifen. When AIs are used in premenopausal women they must be combined with surgical or medical ovarian ablation. Results with AIs in the adjuvant or neoadjuvant setting are detailed below.

### Neoadjuvant therapy with aromatase inhibitors vs. tamoxifen

Systemic treatment administered before definitive surgery is termed neoadjuvant therapy and is often used in women who have clinically involved nodes or a tumour that is ≥ 3 cm. Under these circumstances the chance of occult metastatic disease is high, and the chance of breast conservation with a cosmetically acceptable outcome is low. Neoadjuvant treatment both increases the chance of breast conservation and promotes timely treatment of occult metastases. Pathological response to neoadjuvant chemotherapy is an important prognostic factor. Women with a pathological complete response in breast and lymph nodes to neoadjuvant chemotherapy have as much as a 95%, 5-year distant, disease-free survival (DFS) (19). Although pathological complete response rates after neoadjuvant chemotherapy are in the 20% or higher range for hormone receptor-negative tumours, they are rare with tumours that are hormone receptor-positive.

Neoadjuvant trials with antihormone therapy have generally shown that the chance of breast conservation is higher with AIs than tamoxifen and may be higher for AIs than for chemotherapy in women with hormone receptor positive tumours (19–22). In a trial comparing neoadjuvant letrozole with tamoxifen, the mammographic complete response rate with letrozole, although very low, was still higher than that observed for tamoxifen (20). In the Immediate Preoperative Anastrozole, Tamoxifen or Combined with Tamoxifen trial, women randomised to anastrozole alone were significantly more likely to have experienced sufficient tumour regression to be eligible for breast-conserving surgery than women randomised to tamoxifen or combined treatment (23). Neoadjuvant antihormonal therapy with an AI is a particularly attractive option for postmenopausal women who wish to attempt breast conservation and have strongly ER- and PgR-positive tumours that are ≥ 3 cm and have low proliferation rates.

### Adjuvant therapy with aromatase inhibitors vs. tamoxifen

Clinical trials of AIs as adjuvant therapy have followed one of four approaches: (i) a head-to-head comparison of tamoxifen vs. an AI; (ii) extended adjuvant therapy following initial adjuvant therapy (5 years of an AI after 5 years of tamoxifen); (iii) switching to an AI for 2–3 years after 2–3 years of tamoxifen and (iv) combination therapy using both an AI and tamoxifen simultaneously. All AI approaches except the simultaneous combination of an AI and tamoxifen are associated with fewer breast cancer-related events than tamoxifen alone.

#### Head-to-head comparisons of an aromatase inhibitor and tamoxifen

The Anastrozole, Tamoxifen Alone or in Combination (ATAC) trial randomised more than 9000 women to 5 years of tamoxifen, anastrozole or both agents in combination. The combination treatment did not show a benefit and is not discussed further. Sixty-one per cent of women had no disease detected in their lymph nodes (referred to as node negative) at diagnosis. After 5 years of treatment, there was a significant improvement in DFS in the group of women treated with anastrozole alone regardless of tumour size, nodal status or use of adjuvant chemotherapy before the randomisation. There was a significant interaction with hormone receptor status: women who had ER-positive but PgR-negative tumours were likely to have a superior outcome with anastrozole, whereas women with tumours that were positive for both receptors did just as well with tamoxifen as with anastrozole. The absolute improvement in DFS with 5 years of anastrozole, compared with 5 years of tamoxifen, was 2.5% (p = 0.005). The incidence of contralateral breast cancer was reduced by 53% in women with hormone receptor-positive tumours. No overall survival benefit or significant reduction in deaths from breast cancer was demonstrated for anastrozole in this study. However, there appears to be an emerging survival benefit for women with ER-positive tumours who also had evidence of tumour cells in their draining lymph nodes (referred to as node positive) (24,25).

In the Breast International Group's Femara-Tamoxifen trial, also known as BIG 1–98, 5 years of adjuvant letrozole was compared with 5 years of tamoxifen in postmenopausal women with ER-positive and/or PgR-positive breast cancer. Eventually, this trial was modified with the addition of two treatment groups in which women were either switched from tamoxifen to letrozole or from letrozole to tamoxifen after the initial 2 years of treatment (26). Approximately 8000 patients were randomised to receive tamoxifen or letrozole as their initial therapy. Fifty-nine per cent of women were node negative, and the median age was 61. At a median follow-up of slightly more than 2 years, there was a significant 3.4% absolute improvement in DFS with letrozole compared with tamoxifen. Women with PgR-positive and PgR-negative cancer appeared to benefit equally from letrozole compared with tamoxifen. An approximate 50% reduction in risk of contralateral breast cancer was observed. No significant overall survival benefit was reported, although there was a numeric reduction in deaths from breast cancer and an increase in deaths because of other causes in the group treated initially with letrozole (26). These results were recently updated analysing only those women randomised to 5 years of letrozole vs. placebo. At a median follow-up of 51 months there continues to be a 3% absolute improvement in DFS (18% relative reduction) following letrozole with no improvement in overall survival (27).

The ongoing Tamoxifen Exemestane Adjuvant Multi-institutional (TEAM) trial compares exemestane with tamoxifen as first-line adjuvant treatment. The TEAM trial is designed to compare DFS in patients treated with exemestane vs. tamoxifen at 2.75 years, and to compare DFS in patients treated with 5 years of up-front exemestane vs. tamoxifen for 2.5–3 years followed by 2–2.5 years of exemestane. Enrolment was completed in January 2006 (*n* = 9786). We are awaiting the efficacy results of this trial.

#### Aromatase inhibitors as extended endocrine adjuvant therapy

Given the appreciable late recurrence rates in women with ER-positive breast cancer following 5 years of adjuvant tamoxifen, the MA.17 trial was designed to determine whether 5 years of letrozole (after 5 years of adjuvant tamoxifen) would improve DFS compared with placebo. At a median follow-up of 2.4 years from the time of randomisation, letrozole improved DFS, compared with placebo, by a relative value of 43% and an absolute value of 6%. This was significant regardless of nodal status (28). The trial was unblinded, with women who received placebo given open-label treatment with letrozole on request (28). In an update of this study, a significant reduction in death from any cause was noted for node-positive women receiving letrozole (29). Incidence of menopause-related symptoms, new onset of osteoporosis, arthralgias and alopecia (generally minimal to mild) were all higher for women randomised to letrozole compared with placebo. There was no increase in the rate of bone fracture. There were some specific quality of life domains which were significantly worse with letrozole, including physical functioning, bodily pain, vitality, vasomotor symptoms and sexuality (30).

#### Switching therapy

The switching strategy was designed to: (i) combine the apparent superior efficacy of AIs with tamoxifen's favourable effects on bone and (ii) expose tumour cells to anti-hormonal therapies with two different mechanisms of action. Several adjuvant trials were designed in which, after 2–3 years of adjuvant tamoxifen, women were randomised to continue taking tamoxifen for another 2–3 years or switch to an AI. One such trial, the Intergroup Exemestane Study (IES), randomised 4742 postmenopausal women after 2–3 years of tamoxifen to exemestane 25 mg/day or to continued tamoxifen of sufficient duration to complete a 5-year course of adjuvant therapy (31). Fifty-one per cent of patients were node negative at baseline, and 81% were known to have ER-positive breast cancer. At a median follow-up of 30.6 months, exemestane was associated with a 32% reduction in risk of local or metastatic recurrence, contralateral breast cancer, or death, for an absolute benefit of 4.7% in terms of DFS compared with tamoxifen (31). A recent update at 58 months showed similar improvement in DFS in both the intent-to-treat (24%) and ER-positive/unknown population (26%). A 45% relative reduction in the incidence of contralateral breast cancer was observed. A 17% relative increase in overall survival (p = 0.05) was reported for women randomised to switch to exemestane compared with those remaining on tamoxifen if their tumours were ER-positive or ER unknown (32). Quality of life measured at 3- to 6-month intervals during the first 24 months was similar for women taking exemestane or tamoxifen (33).

In other switching trials, such as the Italian Tamoxifen Arimidex (ITA) trial and the Austrian Breast and Colorectal Study Group 8 (ABCSG 8)/Arimidex-Nolvadex (ARNO 95) combined analysis, switching to anastrozole after 2 years of tamoxifen was compared with continued tamoxifen treatment. A 39% relative improvement in DFS (p = 0.049) and 52% improvement in overall survival were seen at a median follow-up of 30 months in the ABCSG 8/ARNO 95. Improvement in DFS was observed for ITA (34,35).

In summary, all the adjuvant trials in postmenopausal women – whether they involved initial head-to-head comparison with tamoxifen (ATAC, BIG 1–98), switching to an AI after 2–3 years of tamoxifen (IES, ITA and ABCSG 8/ARNO 95), or administering 5 years of an AI after 5 years of tamoxifen – show improvement in DFS favouring the AI. An overall survival benefit is emerging in at least two of the switching trials in women randomised to 2–3 years of an AI following 2–3 years of tamoxifen vs. continuing on tamoxifen (32,35). No significant overall survival benefit has been demonstrated to date for up-front AI administration with letrozole or anastrozole or extended adjuvant therapy with letrozole, although node-positive women appear to show a survival benefit. Follow-up in these trials is short, and an overall survival advantage is likely with up-front AI use. The lack of an early overall survival advantage with AIs in the up-front setting compared with the switch setting may be due to the fact that the switch trials, by excluding women who relapse on tamoxifen in the first 2–3 years, enroll women who are most likely to respond to antihormone therapy. At present, the American Society of Clinical Oncology Technical Assessment recommends that postmenopausal women with receptor-positive breast cancer receive an AI as part of their adjuvant therapy, either as initial therapy, as part of a switching strategy, or after 5 years of tamoxifen (18).

There is no clear advantage to one AI vs. another at the present time. Oncologists often select an AI depending on the type of adjuvant strategy they wish to employ. Several head-to-head trials comparing one AI to another in the adjuvant setting are ongoing. These include trials of anastrozole vs. exemestane and anastrozole vs. letrozole.

## Use of aromatase inhibitors in premenopausal women

Responses have been observed in premenopausal women with concomitant goserelin and AI treatment following tamoxifen failure (36,37). This concept is also being tested in the adjuvant setting with the Suppression of Ovarian Function (SOFT) and Tamoxifen or Exemestane Plus Ovarian Ablation (TEXT) trials. In the SOFT trial, women who are premenopausal after any adjuvant chemotherapy and have ER-positive tumours are randomised to tamoxifen, tamoxifen plus an LHRH analogue or exemestane plus the LHRH analogue (other types of ovarian ablation are also allowed). In the TEXT trial, premenopausal women who may or may not have received chemotherapy are randomised to receive tamoxifen or exemestane, both with an LHRH analogue. The TEXT trial is nearing completion of accrual. It is not clear whether an AI with ovarian ablation will be as good as or better than tamoxifen with or without ovarian ablation at this time. If an AI is given to a premenopausal woman outside of these ongoing trials ovarian ablation with oophorectomy or ovarian suppression with an LHRH analogue must be given. If ovarian suppression with an LHRH analogue is chosen, serum estradiol levels must be monitored regularly to ensure that they remain in the postmenopausal range.

## Adverse event profile of aromatase inhibitors compared with tamoxifen

The adverse event profile for AIs differs from that of tamoxifen. There is no increase in uterine cancers or thromboembolic events as is observed with tamoxifen, but with the exception of hot flushes. Women taking AIs are more likely to complain of symptoms related to estrogen deprivation. Women taking AIs are also more likely to report musculoskeletal adverse events than women taking tamoxifen. These are considered in detail below.

### Gynaecological sequelae

Use of AIs is associated with a higher frequency of vaginal dryness, loss of libido and painful intercourse than is tamoxifen. There are fewer instances of vaginal bleeding and endometrial cancer with AIs than with tamoxifen (30,33,38). AIs are associated with hot flushes, but the proportion of women who exhibit vasomotor instability may be less than that seen with tamoxifen treatment (31,39). Younger age at initiation of treatment is associated with increased frequency of hot flushes (40).

### Musculoskeletal effects

Studies of tamoxifen in postmenopausal women have shown reduction in bone turnover markers and an increase in bone density and the opposite effects with AIs (41–44). These differential effects are not surprising because tamoxifen exerts partial estrogen agonist effects on bone in postmenopausal women, and osteoporosis has been strongly associated with the low serum estrogen levels that occur following AI administration (45). Although a head-to-head comparison of the three third-generation AIs in the Letrozole, Exemestane, Anastrozole Pharmacodynamics study has shown a similar effect on markers of bone turnover for all three drugs (46), it has also been suggested that exemestane may be associated with less of a deleterious effect than is seen with the other third-generation AIs (47). Additional data are expected from a bone substudy in MA.27, an adjuvant trial comparing anastrozole with exemestane.

In adjuvant studies, all three third-generation AIs – anastrozole, letrozole and exemestane – have shown an increased risk of bone fracture compared with tamoxifen. The absolute differences, while statistically significant in the ATAC trial of anastrozole vs. tamoxifen and the BIG 1–98 trial of letrozole vs. tamoxifen, were only 1–4%. Most fractures were in the spine and not the hip (27,48). The difference in fracture rate approached, but did not reach, statistical significance in the IES trial (3.1% for women switching to exemestane vs. 2.3% in women continuing on tamoxifen) (31). Letrozole given in MA.17 after 5 years of tamoxifen had a numerically higher fracture rate than placebo (5.3% vs. 4.3%), but like the IES trial, the absolute excess fracture rate was ≤ 1% and statistically insignificant (43). This would seem to indicate that tamoxifen taken before an AI provides some measure of bone mineral density protection in postmenopausal women.

Bisphosphonates can be used to prevent the bone mineral loss observed with AIs. This strategy was successfully used in the Zometa-Femara Adjuvant Synergy trials, and the Austrian Breast and Colorectal Cancer Study Group trial 12, in which an intravenous bisphosphonate, zoledronic acid, was administered every 6 months for the duration of AI therapy (49,50). Vitamin D supplementation is advisable in women with serum 25-OH vitamin D levels < 30 ng/ml because women with baseline vitamin D insufficiency are at an increased risk of bone loss when receiving AIs (51).

In randomised studies, arthralgias/myalgias have been reported significantly more frequently in women randomised to AIs than in those randomised to tamoxifen or placebo. The absolute frequency varies tremendously from trial to trial (5.4–37% for AIs vs. 3.6–26% for tamoxifen or placebo), which in turn probably reflects the method used to record the symptoms. The incidence of arthralgias and myalgias appear to be about two-thirds higher with an AI than with tamoxifen or placebo but usually improves with time (38). Two small studies have shown that women taking AIs for cancer therapy often have deficient or suboptimal 25-OH vitamin D levels in their serum (51,52). Improvements in myalgias and arthralgias were observed in a high proportion of women with deficient or suboptimal levels of vitamin D who were given prescription-strength vitamin D for 12 weeks (52). Serum 25-OH vitamin D is used to assess adequacy of total body vitamin D stores (53) and levels should be checked prior to starting AI treatment to make sure they are in the optimal range of 30–50 ng/ml (53–55). In general, each additional 1000 IU of vitamin D3 can be expected to increase 25-OH-D serum levels by 10 ng/ml. The addition of celecoxib 400 mg bid to exemestane reduced arthralgias and improved response rates in a placebo-controlled trial in women with metastatic disease (56). Prospective trials are under way to assess the prevalence of vitamin D deficiency in women undergoing adjuvant therapy with AIs, correlation with the development of myalgias/arthralgias and the relief of symptoms with vitamin D replacement.

### Thromboembolic and cardiovascular effects

Aromatase inhibitors do not increase the risk of deep venous thrombosis; this differs from tamoxifen, for which the risk of deep venous thrombosis and pulmonary embolism is increased approximately twofold (57,58). Further, except for a higher frequency of occurrence in women over 50 and those with high body mass index, there does not appear to be an easily identified predisposing factor behind the majority of episodes of deep venous thrombosis associated with tamoxifen (59).

Aromatase inhibitors in adjuvant trials have been associated with an increase in ischaemic cardiovascular events and a numeric, but not statistically significant increase in cardiac deaths when compared with tamoxifen (25,30,31), but not when compared with placebo (29). AIs do not have a substantial effect on lipid metabolism (39,58). It is possible that, if there is an intrinsic adverse effect of AIs on ischaemic heart disease, it might be due to estrogen depletion in the coronary arteries leading to loss of the vasodilatory response of estrogen to stress (60). Alternatively, the observation might stem from a small cardio-protective benefit from tamoxifen rather than a deleterious effect of AIs. With the exception of triglycerides, tamoxifen has a favourable effect on the serum lipid profile (1) and tamoxifen has also been observed to improve endothelial function and reduce carotid intima–media thickness in postmenopausal women (61). Despite tamoxifen's favourable effects on some lipid and endothelial parameters, there is as yet no conclusive evidence that tamoxifen exhibits cardioprotective effects (62). The lack of significant cardiovascular benefit in most randomised trials for tamoxifen may be due to an increase in triglycerides and clot promoting proteins, which offset the beneficial cardiovascular effects of tamoxifen (1,59). An additional factor might be the widespread use of statins, which would obscure tamoxifen's favourable effects on cholesterol. In the ATAC trial, 4.1% of participants randomised to anastrozole vs. 3.4% of those randomised to tamoxifen died from ischaemic heart disease (25). In the IES trial, at 3-year follow-up, a higher number of cardiovascular deaths were reported for exemestane than for tamoxifen (1.1% vs. 0.8%) (31). In the BIG 1–98 trial, 2.5% of women randomised to letrozole had serious or fatal cardiac events compared with 1.1% taking tamoxifen; this was highly significant (27). There were also twice as many cardiac deaths with letrozole than with tamoxifen (13 vs. 6), but given the small number of events, the difference was not statistically significant.

Because the proportional differences in cardiac deaths observed in women randomised to AI vs. tamoxifen are < 1%, a potential increase in cardiovascular events is not likely to be a major concern for women undergoing cancer therapy with an AI. However, enthusiasm for AI use in the primary prevention setting will be limited if AIs are found to be associated with a higher number of cardiac events compared with placebo or tamoxifen.

## Management and prevention of adverse events

As AI use becomes more common, internists will undoubtedly be asked by their patients for help with management and prevention of adverse events, although the relative risks and benefits of AIs vs. other hormonal therapy will hopefully have been discussed by the patient's oncologist.

For vasomotor symptoms, non-hormonal methods such as selective serotonin reuptake inhibitors (SSRIs), gabapentin or clonidine should be tried first (63). In doses commonly needed for relief of hot flushes (75 mg venlafaxine, 20 mg fluoxetine and 300–900 mg gabapentin), side effects for these medications include drowsiness, dry mouth and dyspepsia. Use of SSRIs may also contribute to the loss of sexual interest.

Vaginal dryness that is not ameliorated with lubricants may be treated with poorly absorbed vaginal estrogens, such as oestradiol vaginal rings or tablets. However, a small study showed a significant increase in serum estrogen levels following use of these preparations (64). A weak preparation (1%) of testosterone with 2 mg of estriol (1 g administered 2–3 times weekly) is often effective for improving vaginal dryness, dyspareunia and libido. When women are taking AIs, testosterone cannot be readily converted to estradiol. Estriol is a very weak estrogen and likewise cannot be converted to estradiol (65). There is little information regarding the safety of this practice, particularly in women with prior breast cancer (66).

## Aromatase inhibitors for breast cancer prevention

Tamoxifen fails to prevent ER-negative breast cancer, and one-third or more of ER-positive breast cancers (67–70). The incomplete efficacy, increased risk of serious adverse events, and the lack of survival benefit with tamoxifen given as primary prevention (66–70) fuels the effort to develop safer and more effective primary-prevention strategies. The superior DFS observed for AIs compared with tamoxifen in the adjuvant setting combined with the lack of increase in thromboembolic events or uterine cancer has led to the initiation of multiple primary-prevention trials in high-risk women without prior breast cancer. Currently, there are several major multi-institutional primary-prevention trials in postmenopausal women in which an AI is being compared with placebo (Table 2).

**Table 2:** Ongoing Multi-institutional Phase III Primary Prevention trials of AIs in postmenopausal women

Trial	Agents studied	Duration studied (years)
International Breast Cancer Intervention Study II	Anastrozole vs. placebo	5
Aromasin Prevention Study	Exemestane vs. placebo	3
National Cancer Institute of Canada Clinical TrialsGroup MAP.3 Breast Cancer Prevention Trial	Exemestane vs. placebo	5

AIs, aromatase inhibitors.

Of serious concern for prevention is the potential for increase in risk of bone fracture and cardiovascular disease related to long-term estrogen depletion with AIs. However, arthralgias, fatigue, dyspareunia, reduced libido and hot flushes may result in poor uptake and/or compliance. Ongoing phase III prevention trials will define the incidence of these adverse events relative to placebo in a healthy population, and potential solutions to avoid some of these problems in the prevention setting are already being explored.

One small study indicates that bone mineral loss after AIs is primarily limited to women with insufficient 25-OH vitamin D levels (71). Given the importance of adequate vitamin D in health, practitioners should strive to achieve 25-OH vitamin D levels of at least 30 ng/ml (55,72). Bisphosphonates have been found effective in preventing AI- and cancer-therapy-related bone mineral loss in the adjuvant setting (73,74). Along with exercise and appropriate supplementation of calcium and vitamin D, bisphosphonates could be used along with AIs to prevent bone loss. Very low-dose estradiol (0.015 mg estradiol patch replaced twice weekly) increased serum estradiol to a median of 12 pmol and may be effective in reducing the increased bone turnover associated with AI use (75).

Statins could be used along with AIs to improve both lipid profiles and endothelial function. There is also a suggestion that long-term use of a lipophilic statin might reduce breast cancer risk (76), but results in case–control studies are mixed (77–79). However, because both statins and AIs are metabolised in the liver, pharmacological and pharmacodynamical studies need to be completed to better understand how concomitant administration might affect levels of both drugs.

Approximately one-quarter of perimenopausal and postmenopausal women take hormone replacement therapy for some period of time during menopause or menopause transition (80). Although other drugs give partial relief of symptoms associated with the climacteric, none is as effective as hormone replacement (81). The Women's Health Initiative (WHI) indicates a nonsignificant increase in the risk of breast cancer and coronary heart disease for women taking combined oral equine estrogen plus a progestin after 5 years. However, there was no increase in breast cancer risk in the WHI for women taking estrogen alone at a median follow-up time of ∼7 years (82,83). In fact, updated results indicate that women aged 50–59 randomised to estrogen alone had a nonsignificant reduction in breast cancer and coronary heart disease. Further, for women aged 50–59 randomised to estrogen alone or combined estrogen plus progestin there was a significant 30% reduction in overall mortality compared with those randomised to placebo (84,85). The Million Women Study showed a modest increase in risk of breast cancer for hormone replacement therapy given by any route with the exception of vaginal hormones. Similar to the WHI, women taking estrogen and a progestin had a higher relative risk than those receiving estrogen alone (86). Few prevention options are available for those women who need hormone replacement for the management of menopausal symptoms and who are at increased risk for breast cancer because of family history or other factors. Tamoxifen and hormone replacement (usually transdermal) are commonly prescribed together in Europe, but this is generally not performed in the USA (87). Furthermore, updated analyses of the three major primary prevention trials of tamoxifen vs. placebo in which hormone replacement was allowed have yielded conflicting results. The Italian prevention trial conducted predominately in average risk hysterectomised women found a reduced risk of ER+ breast cancer with tamoxifen only in those women at increased risk because of hormone replacement or other factors (69). The Royal Marsden trial showed a reduced incidence of ER+ breast cancer whether women took hormone therapy or not. However, the International Breast Cancer Intervention Study 1 trial results indicated tamoxifen was not effective in women beginning hormone replacement therapy (HRT) during study (68–70).

Preclinical studies indicate that AIs might be effective in reducing the risk of breast cancer in hormonally intact animals under circumstances in which breast aromatase is up-regulated (88). In studies of postmenopausal women, breast estradiol levels have been found to be 10- to 50-fold higher than serum levels, and aromatase – which is up-regulated in proliferative breast disease – is responsible for much of this local synthesis (89,90). We have performed a 6-month pilot study of letrozole in high-risk women who continued to take their hormone replacement during the study period. An approximate two-thirds reduction in breast tissue proliferation (Ki-67) was observed after 6 months of letrozole. There was no increase in hot flushes or arthralgias for the majority of women in the trial (91). The concept of using an AI in women already receiving hormone replacement therapy will be explored further in a placebo-controlled, randomised, proof-of-principle trial in which change in Ki-67 in benign breast tissue is the primary end-point. In this ongoing study, the change in bone turnover markers and the cardiovascular risk biomarkers will also be explored.

## Overcoming resistance to aromatase inhibitors

Even with an initial response to treatment, for women with metastatic disease, resistance eventually develops to AIs and clinical regrowth of tumour is observed. In most cases, the resistant cancer continues to be ER positive. There are several mechanisms of resistance demonstrated in animal models. These include: (i) development of hypersensitivity of the ER to very low levels of estrogen; (ii) up-regulation of growth factor receptors and/or associated signalling pathways (HER-2, EGFR and insulin growth factor receptor (IGFR)) (92,93). Reduction in the level of ER expression would theoretically reduce the sequelae of ER hypersensitivity and could be accomplished by increasing ER ubiquitisation with a drug such as fulvestrant, an ER down-regulator (94). Results from animal models suggest that the AI letrozole plus fulvestrant is more effective than either alone (93). Fulvestrant is often effective as antihormonal therapy following response and progression on an AI and is equally effective as anastrozole in women with metastatic disease who have previously been treated with tamoxifen (94,95). Fulvestrant plus anastrozole is currently being compared with anastrozole alone in metastatic disease in the co-operative group setting. The use of short courses of physiological or pharmacological doses of estradiol to induce apoptosis in breast cancer cells with a hypersensitive ER in women whose tumours are resistant to multiple types of endocrine therapy including AIs has been suggested based upon preclinical models (96,97). Combination regimens of AIs and several types of growth factor receptor or activated pathway inhibitors are being explored (98–100).

## Summary

The third-generation AIs are now preferred therapy for postmenopausal women with hormone receptor-positive tumours in both the early and metastatic settings. Switching from adjuvant tamoxifen to an AI (exemestane or anastrozole) after 2–3 years of tamoxifen has shown superior DFS and overall survival compared with continuing on tamoxifen. Using anastrozole or letrozole instead of adjuvant tamoxifen as initial therapy (with or without prior adjuvant chemotherapy) has also shown superior DFS. Finally, for women completing 5 years of tamoxifen, extended adjuvant antihormonal therapy with letrozole has shown a reduced recurrence rate, particularly for node-positive patients. American Society of Clinical Oncology guidelines recommend that an AI be included in a woman's adjuvant regimen if she has ER-positive and/or PgR-positive breast cancer. The decision to use AI as initial endocrine therapy, as opposed to switching to an AI after 2–3 years of tamoxifen therapy, is likely to be guided by the tumour characteristics. Patients who have ER-positive tumours with unfavourable characteristics, such as HER-2 positivity, PgR negativity or nodal positivity, are likely to be selected for immediate AI therapy. However, patients with ER-positive tumours without unfavourable characteristics are likely to be selected for tamoxifen treatment for 2–3 years before taking an AI for 2–3 years. Several ongoing clinical trials are examining the use of AIs in women at an elevated risk of developing breast cancer. Critical to the ultimate success of AIs in both the adjuvant and preventive settings will be management of adverse events, particularly bone mineral density loss, arthralgias and gynaecological sequelae.
